# Predicting the Influence of Axon Myelination on Sound Localization Precision Using a Spiking Neural Network Model of Auditory Brainstem

**DOI:** 10.3389/fnins.2022.840983

**Published:** 2022-03-14

**Authors:** Ben-Zheng Li, Sio Hang Pun, Mang I. Vai, Tim C. Lei, Achim Klug

**Affiliations:** ^1^Department of Physiology and Biophysics, University of Colorado Anschutz Medical Campus, Aurora, CO, United States; ^2^Department of Electrical Engineering, University of Colorado, Denver, Denver, CO, United States; ^3^State Key Laboratory of Analog and Mixed Signal Very-Large-Scale Integration (VLSI), University of Macau, Taipa, Macau SAR, China; ^4^Department of Electrical and Computer Engineering, Faculty of Science and Technology, University of Macau, Taipa, Macau SAR, China

**Keywords:** sound localization, auditory brainstem, medial superior olive, myelin alteration, interaural time difference, spiking neural network, computational model

## Abstract

Spatial hearing allows animals to rapidly detect and localize auditory events in the surrounding environment. The auditory brainstem plays a central role in processing and extracting binaural spatial cues through microsecond-precise binaural integration, especially for detecting interaural time differences (ITDs) of low-frequency sounds at the medial superior olive (MSO). A series of mechanisms exist in the underlying neural circuits for preserving accurate action potential timing across multiple fibers, synapses and nuclei along this pathway. One of these is the myelination of afferent fibers that ensures reliable and temporally precise action potential propagation in the axon. There are several reports of fine-tuned myelination patterns in the MSO circuit, but how specifically myelination influences the precision of sound localization remains incompletely understood. Here we present a spiking neural network (SNN) model of the Mongolian gerbil auditory brainstem with myelinated axons to investigate whether different axon myelination thicknesses alter the sound localization process. Our model demonstrates that axon myelin thickness along the contralateral pathways can substantially modulate ITD detection. Furthermore, optimal ITD sensitivity is reached when the MSO receives contralateral inhibition via thicker myelinated axons compared to contralateral excitation, a result that is consistent with previously reported experimental observations. Our results suggest specific roles of axon myelination for extracting temporal dynamics in ITD decoding, especially in the pathway of the contralateral inhibition.

## Introduction

In the mammalian brain, the precise temporal information encoded in action potentials and trains of action potentials is one major mechanism contributing to accurate neural integration and information processing. Such temporal precision is especially crucial for the localization process of sound sources in the auditory brainstem, especially for low-frequency sound sources. This process works through the microsecond-precise integration of signals between the two ears such that whenever the temporal precision is even slightly altered, sound localization accuracy suffers significantly (reviewed in Grothe, 2003; Tollin and Yin, 2009; Grothe et al., 2010). Myelination of afferent fibers is a key mechanism in mammalian brains to ensure reliable, energy-efficient and temporally precise action potential propagation, and therefore, myelination is tightly controlled and actively managed in the brain (reviewed in Debanne, 2004). It is therefore not unexpected that such mechanisms have been described in the sound localization pathway as well (Ford et al., 2015; Seidl and Rubel, 2016; Stange-Marten et al., 2017).

In the auditory brainstem, sound localization along the azimuth is accomplished by the two principal localization nuclei, the lateral and the medial superior olive (LSO and MSO, respectively). High-frequency sounds are localized in the LSO by calculating the interaural level difference (ILD; Boudreau and Tsuchitani, 1968), while low-frequency sounds are localized in the MSO by calculating the interaural time difference (ITD; Goldberg and Brown, 1969). Mammals, including human listeners, are typically capable of resolving two sound sources that are just a few degrees separated from each other, by resolving ITDs as small as several microseconds (Grothe et al., 2010). How exactly the sound localization circuit can accomplish this extraordinary computational result within a set of fixed exterior constraints has been the subject of a number of studies. The speed of sound in air, as well as an animal’s head size, dictate particular ITDs. For different species with different head sizes, the relationship between ITD and corresponding spatial angle varies, but for most species and most spatial angles, ITDs are one to two orders of magnitude shorter than the duration of an action potential. To operate at such temporal precision, several neural mechanisms along the afferent pathways have been described, including kinetically fast ion channels, large and electrically compact synapses, tuning of cochlear, synaptic, post-synaptic, and transmission delays (reviewed in Trussell, 1999; Grothe, 2003; Tollin and Yin, 2009; Grothe et al., 2010). Some of these mechanisms remain plastic during an individual’s lifetime, allowing for a recalibration of the network, for example in response to hearing loss. Other mechanisms, such as axon myelination, are plastic during a period in which the animal’s head is still growing and thus ITDs are changing, but do not re-calibrate after adulthood (Sinclair et al., 2017). Once calibrated, the various inputs to MSO neurons show different axon myelination patterns, which precisely preserve but also actively control the timing of action potentials propagated in these afferents, thereby controlling the sound localization process at MSO neurons.

In this study, we investigate contralateral excitatory and inhibitory inputs to the MSO, which show different axon myelination patterns (Morest, 1968; Grothe et al., 2010). The contralateral inhibitory pathway consists of axons with thicker layers of myelin, resulting in higher conduction velocities, compared to those of the ipsilateral excitatory pathway. Experimental results suggest that axonal myelination may be specifically adapted for tuning the input timing to the MSO, thereby actively contributing to spatial hearing perception (Schwartz, 1992; Ford et al., 2015; Seidl and Rubel, 2016; Stange-Marten et al., 2017). However, axon myelination as a factor in circuit modeling is underexplored and is simply included as a constant in most models—probably due to our still incomplete understanding of the structure-function relationships (Nave, 2010). In this study, the role of myelin morphology as a contributing factor to the MSO sound localization circuit is specifically explored.

Prior modeling efforts of ITD coding at MSO primarily focused on the effects of post-synaptic integration of MSO neurons (Brand et al., 2002; Zhou et al., 2005; Leibold, 2010; Brughera et al., 2013; Myoga et al., 2014). Other studies modeled the axonal propagation time as a constant delay, not including axonal morphology (Wang et al., 2014; Encke and Hemmert, 2018). On the other hand, some studies included action potential timing difference by varying axonal propagation delays (Glackin et al., 2010; Pan et al., 2021). These models, however, were based on the Jeffress model of a delay line structure (Jeffress, 1948), which is anatomically inconsistent with neural inhibition observed in mammalian ITD circuits (Brand et al., 2002; Grothe and Pecka, 2014; Franken et al., 2015).

This study employed a spiking neural network (SNN) model to investigate how axonal structure and synaptic adaptation between the excitatory and inhibitory inputs to MSO can affect ITD decoding. Specifically, the myelination thickness and the synaptic conductance were modeled in detail and compared with pure tone and natural sound stimulation regarding ITD coding accuracy and minimum temporal discrimination. Based on this SNN model and our decoding analysis, we found that the axon myelination patterns of both contralateral excitatory and inhibitory pathways can significantly modulate ITD decoding. The variation of myelin thickness, which results in conduction velocity variations along the excitatory pathways, can significantly shift the ITD tuning curve. On the other hand, axonal myelination and synaptic strength variations on the inhibitory pathway can significantly influence ITD sensitivity and precision.

## Materials and Methods

### Neuron and Synapse Model

The spiking neurons were modeled under a conductance-based leaky integrate-and-fire scheme. The membrane potential (*v_m*) of a spiking neuron was described by the following first-order differential equation:


$$C_{m}\,\frac{d\,v_{m}\,\left(t\right)}{d\,t}=g_{l}\,\left(E_{l}-v_{m}\,\left(t\right)\right)+I_{s\,y\,n}\,\left(t\right),$$

where *I*_*syn*_ is the total synaptic current comprising an excitatory and an inhibitory component:


$$I_{s\,y\,n}\,\left(t\right)=g_{e}\,\left(t\right)\,\left(E_{e}-v_{m}\,\left(t\right)\right)+g_{i}\,\left(t\right)\,\left(E_{i}-v_{m}\,\left(t\right)\right),$$

$$\tau_{e}\,\frac{d\,g_{e}\,\left(t\right)}{d\,t}=-g_{e}\,\left(t\right),$$

$$\tau_{i}\,\frac{d\,g_{i}\,\left(t\right)}{d\,t}=-g_{i}\,\left(t\right).$$

Descriptions of the parameters and the values used in the simulation are listed in Table 1. The excitatory and inhibitory synaptic conductances were modeled as first-order time decaying parameters with lifetimes of τ_*e*_ and τ_*i*_. When an action potential arrived at the presynaptic membrane, the conductance increased by Δ*g*_*exci*_ or Δ*g*_*inhi*_, and subsequently decayed as described by the time constants. The spiking neuron will elicit an action potential when the membrane potential *v*_*m*_(*t*) reaches the firing threshold *V*_*th*_, and the membrane potential *v*_*m*_(*t*) is then reset to the resting potential *V*_*reset*_ after a short refractory period τ_*ref*_.

**TABLE 1:** List of parameters for neurons and synapses.

Neuron parameter	Value	Description	Synapse parameter	Value	Description
*C_m*	70 pF	Membrane capacitance	*E_e*	0 mV	Excitatory reversal potential
*V*_*th*_	−50 mV	Threshold potential	*E_i*	−70 mV	Inhibitory reversal potential
*V*_*reset*_	−55.8 mV	Reset potential	Δ*g*_*exci*_	15 nS	Excitatory postsynaptic conductance increment
τ_*ref*_	5 ms	Refractory period	Δ*g*_*inhi*_	75 nS	Inhibitory postsynaptic conductance increment
*E_l*	−55.9 mV	Leaky reversal potential	τ_*e*_	0.23 ms	Excitatory time constant
*g_l*	13 nS	Leaky conductance	τ_*i*_	2 ms	Inhibitory time constant

### Network Architecture

**FIGURE 1:**
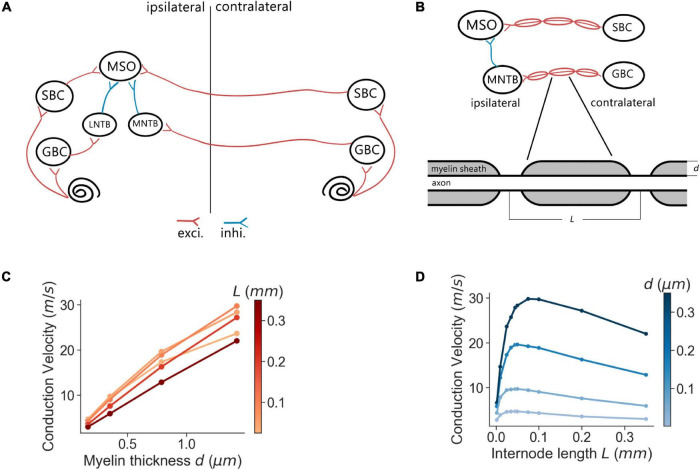
MSO model and myelination patterns. **(A)** Model architecture. **(B)** Myelination patterns along the contralateral pathway to MSO. **(C,D)** Axonal conduction velocity vs. myelin thickness **(C)** and internode lengths **(D)**.

The architecture of the proposed SNN sound localization model (Figure 1A) consists of a left and a right MSOs and related afferent nuclei, including cochlear nucleus and trapezoid body. The cochlea first encodes acoustic stimuli, which are then sent as action potentials into the model through the auditory nerve (AN) and received by the Cochlear Nuclei (CN). At the CN, the Spherical Bushy Cells (SBCs) innervate the two MSOs bilaterally, and Globular Bushy Cells (GBCs) innervate the contralateral Medial Nucleus of the Trapezoid Body (MNTB) as well as the ipsilateral Lateral Nucleus of the Trapezoid Body (LNTB). Under this architecture, MSO cells receive bilateral excitation from the SBCs, ipsilateral inhibition from the LNTB and contralateral inhibition from the MNTB.

Every neural population (AN, GBC, MSO, etc.) in each hemisphere contained 1,000 spiking neurons that stochastically connected to neurons in other populations based on a connection probability (*p*_*connect*_). This stochastic connectivity introduced heterogeneity of input sources across all modeled neurons and rich diversity of neural responses, which was comparable to biological neural circuits. The synaptic connections were also associated with an axonal transmission delay (*t*_*trans*_) and a synaptic delay (*t*_*syn*_). These delays were normally distributed with a standard deviation of 0.05 ms. The transmission delays of the connections that traveled across the midline (SBC to contralateral MSO and GBC to contralateral MNTB) were derived from the corresponding conduction velocities and myelin thicknesses. The SNN model was implemented using the Brian2 simulator (Stimberg et al., 2019) and was simulated on a supercomputing cluster (RMACC Summit, University of Colorado Boulder). The simulation was repeated twenty times with different random seeds to compensate for random effects induced during stimuli encoding, network building, and decoding. Averaged metrics from all random permutations are reported as results. The specific parameters of the connections were adopted from previous studies (Brand et al., 2002; Spirou et al., 2005; Couchman et al., 2010; Roberts et al., 2013, 2014; Encke and Hemmert, 2018) and are listed in Table 2.

**TABLE 2:** List of parameters for neurons and synapses.

Population	Type	Connection	*p*_connect_	${\bar{t}}_{\text{trans}}$ (ms)	${\bar{t}}_{\mathrm{syn}}$ (ms)
AN	Spike generator	AN → ipsi. GBC	4%	0.2	0.6
		AN → ipsi. SBC	4%	0.2	0.6
SBC	Excitatory	SBC → ipsi. MSO	0.6%	0.6	0.6
		SBC → cont. MSO	0.6%	*various^b^*	0.6
GBC	Excitatory	GBC → ipsi. LNTB	0.3%	0.3	0.3
		GBC → cont. MNTB*^a^*	one-to-one	*various^c^*	0.2
MNTB	Inhibitory	MNTB → ipsi. MSO	0.3%	0.1	0.3
LNTB	Inhibitory	LNTB → ipsi. MSO	0.3%	0.1	0.3
MSO	Excitatory				

*^a^The Calyx of Held withΔg_inhi_ = 250 Ns.*

*^b^Depends on the SBC myelin thickness d_SBC_.*

*^c^Depends on the GBC myelin thickness d_GBC_.*

### Stimulus Encoding

For sound stimulation to the cochlea, two spike generators were used at the left and right AN to encode the sound signals into cochlear action potential responses. For pure tone stimulation, sinusoidal sound waves of 300 Hz with a 50 dB sound pressure level (SPL) were used, except where otherwise indicated. The envelope duration of the sinusoidal wave was 100 ms with 20 ms ramp-up and ramp-down periods and sampled at 100 kHz. For natural sound stimulation, sound samples with dominant frequencies ranging from 318 to 546 Hz were created based on 60 bird song clips of the long-eared owl collected from the Xeno-canto project.^1^ The bird song clips were adjusted to 50 dB SPL with 20 ms ramp-up and ramp-down, and were up-sampled from 44.1 to 100 kHz. Much of the previously published physiological data which informed our model were recorded in Mongolian gerbils. Thus, vocalizations of an owl species—a predator of this species—seemed appropriate.

Acoustic stimuli were sent to two ears with ITDs ranging from −1 to +1 ms with step sizes in log-scale. Although the simulated ITD range was beyond the biologically relevant ITD range of gerbils, typically ± 130 μs, this exceeded range was chosen to increase the comparability of our simulated results to existing physiological recordings using these broader ITD ranges (Pecka et al., 2008; Franken et al., 2015). ITD is defined as the onset time difference of the same sound between the left and the right ear, with positive ITDs defined as sound leading at the right ear. For each ITD, the stimulation was repeated ten times for pure tones and eight times for natural sounds. A peripheral hearing model (Zilany et al., 2014; Rudnicki et al., 2015) was used to generate action potentials from the sound waves for the AN spike generators. The simulated AN was configured as a composition of 60% high spontaneous firing rate fibers, 20% medium spontaneous firing rate fiber, and 20% low spontaneous firing rate fibers.

### Axon Myelination and Conduction Velocity

The conduction velocity of myelinated axons was computed using the multi-compartment axon model of Halter and Clark (1991) following the methods and parameters proposed by Ford et al. (2015) for simulating GBC and SBC fibers. Briefly, axons were compartmentalized into nodes and internodes represented by Hodgkin-Huxley type differential equations, which describe the axial current flow in accordance with the kinetics of the inactivating sodium, low-threshold potassium, and leak channels. In the simulation, action potentials were elicited at the first node of the axon by brief current stimulation, and the traveling time of the action potential across twenty internodes was used to calculate the conduction velocity.

The simulations were implemented with the myelinated axon model (Arancibia-Cárcamo et al., 2017)^2^ in MATLAB. In the simulations, the axon internodal length was 0.187 mm (the average of SBC and GBC fibers measured in Ford et al., 2015) except where specified otherwise, and the node diameter was defined as 60% of the axon diameter (Figure 1B). Meanwhile, the myelin thickness of the axon varied from 0.2 to 0.6 μm. The anatomical arrangement of the model was based on adult Mongolian gerbil with stable myelination (Seidl and Rubel, 2016; Sinclair et al., 2017). The axon length from the SBC in CN to MSO on the ipsilateral side and from MNTB to MSO were assumed to have a length of 4.5 mm estimated from a gerbil brain atlas (Radtke-Schuller et al., 2016). As previously reported (Ford et al., 2015), the internodal length decreased at a distance of more than 0.5 mm from the branching area in MSO and 0.7 mm from the heminode near MNTB. At that point, the conduction velocity became more uniform along the rest of the axon, and the transmission delay was directly computed from the conduction velocity and the corresponding axon length with the uniform internodal length, i.e., 4 mm for the SBC-MSO projections and 3.8 mm for the GBC-MNTB projections.

### Data Analysis

**FIGURE 2:**
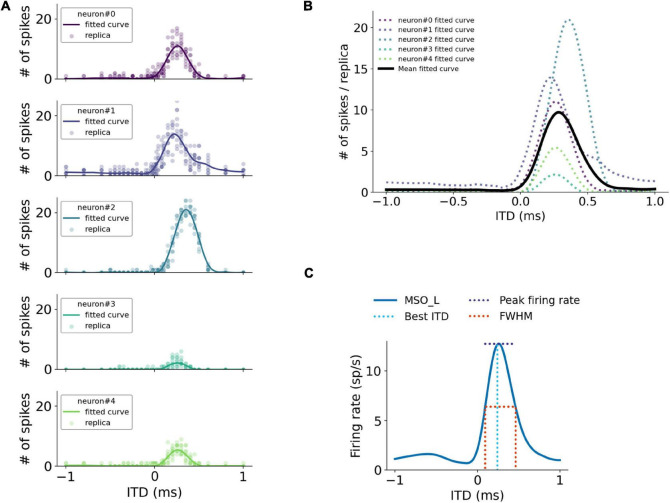
Quantification of simulated MSO responses to ITDs. **(A)** ITD functions of single MSO neurons calculated by curve fitting. **(B)** Overall MSO response to ITDs (black curve) estimated by taking average of single-neuron ITD functions listed in **(A)**. **(C)** Annotation of metrics used for quantifying MSO responses.

ITD responses to various sound stimuli of each MSO neuron were quantified as the firing rates in response to these stimuli (Figure 2A). The overall ITD tuning curves of the MSO were computed as the mean of single-neuron ITD responses in the MSO population trimmed by firing rates between 20 and 80% to omit non-responding neurons (Figure 2B). For a more accurate peak firing rate analysis, the ITD tuning curves were interpolated with a precision of 0.1 μs and subsequently smoothed by a Savitzky–Golay filter with an 80 μs smoothing window. After the ITD curves were smoothed, the peak firing rate positions were then used to define the best ITD. The peak amplitude and the Full-Width-at-Half-Maximum (FWHM) are illustrated in Figure 2C.

For the ITD decoding analysis, the spike counts of MSO neurons during the course of the stimuli were used as decoding features. ITDs were predicted using a Support Vector Machine (SVM) classifier with a linear kernel trained with the leave-one-out cross-validation approach. Three hundred MSO neurons were then randomly selected from both left and right MSO and sent to the SVM classifier to predict ITD in response to each stimulation. The decoding accuracy and the mean squared error (MSE) were determined from the predicted ITDs with 17 ITD sub-classes in the range from –1 to 1 ms. The classification accuracy between 10 and –10 μs ITDs was also computed in the same manner to estimate the precision of the ITD detection.

The sensitivity of ITDs was accessed using the Just-Noticeable-Difference (JND) that quantifies the smallest perceptible change. It was computed by comparing ITD responses symmetrical to the zero time, e.g., –50 and 50 μs ITDs. For each pair of symmetrical ITD responses, the difference of firing rates between left and right MSO were compared using the one-tailed Mann-Whitney *U*-test. The smallest symmetrical ITD that reached the minimum significant level indicates the JND.

### Data Accessibility

The implementation source code and natural sound clips are available on the GitHub repository.^3^

## Results

### Conduction Velocity Varies With Axon Myelination Patterns

We first assessed the correlation between axon myelination patterns and conduction velocity. Our overall results indicate that both the myelin thickness and the internodal length affect conduction velocity. While the level of myelin is directly proportional to the conduction velocity (Figure 1C), the internodal length has a non-linear relationship with the conduction velocity (Figure 1D). Theoretically, increasing the myelin thickness should increase the axial current flow, which in turn increases the propagation speed of action potentials. On the other hand, an increased internodal length results in a greater myelin coverage of the axon, which also increases the conduction velocity. In addition, fewer nodes result in less frequent regeneration of the action potential, additionally speeding up conduction. When the internodal length becomes even longer, the conduction velocity decreases since the transfer efficiency of the depolarization between nodes is lower (Brill et al., 1977; Ford et al., 2015). For the following results, we used the myelin thickness as the major variable for the comparison between different myelinated fibers, while the internodal length was set as a constant for comparison and simplification purposes.

### Spherical Bushy Cell Axon Myelin Thickness and Interaural Time Difference Tuning

**FIGURE 3:**
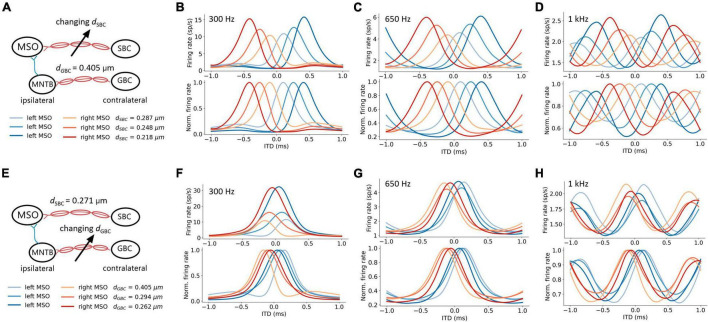
Axon myelination along contralateral pathways influence ITD tuning. **(A–D)** Effects of changing SBC axon myelin thickness. **(E–H)** Effects of changing GBC axon myelin thickness. **(A,E)** Schematic of tuned parameter and legends of ITD tuning curves. **(B–D,F–H)** ITD tuning curve (top), and normalized ITD tuning curve (bottom). **(B,F)** 300 Hz 50 dB SPL pure tone stimulation. **(C,G)** 650 Hz 50 dB SPL pure tone stimulation. **(D,H)** 1 kHz 50 dB SPL pure tone stimulation.

**FIGURE 4:**
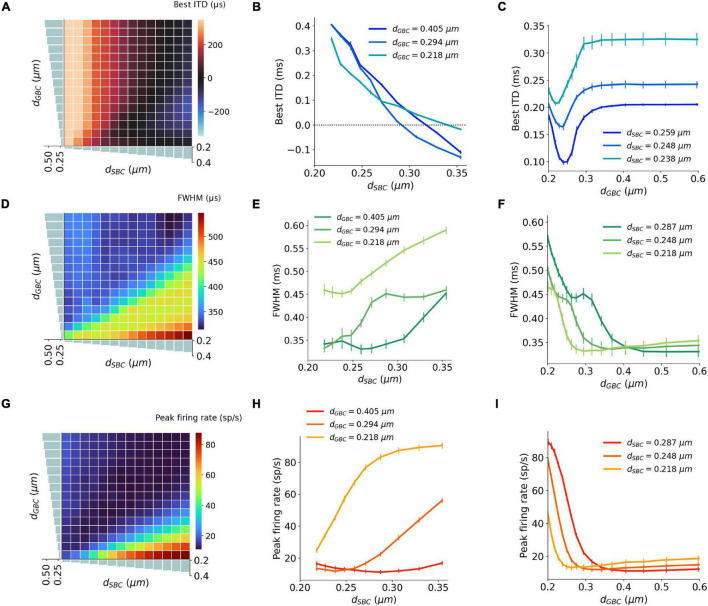
Interaction between SBC and GBC axon myelination on ITD tuning, tested at 300 Hz. **(A–C)** Best ITD as a function of different SBC and GBC myelin thicknesses. **(D–F)** FWHM as a function of different SBC and GBC myelin thicknesses. **(G–I)** Peak firing rate as a function of different SBC and GBC myelin thicknesses. Error bars represent standard errors.

The firing rates of MSO neurons were studied during pure tone sound wave stimulation with varying ITDs, and were additionally recorded against changing myelin thicknesses of the SBC axon (*d*_*SBC*_) along the contralateral excitatory pathway (Figure 3A). The ITD tuning curve shifted toward the center (0-ITD) when SBC myelination increased (Figures 3B–D). The observed shift was qualitatively the same for different sound frequencies (Figures 3B–D) but note that the absolute firing rate decreased with increasing frequency. This decrease is consistent with the reported frequency-dependent thresholds in MSO neurons (Remme et al., 2014; Mikiel-Hunter et al., 2016) and is most likely due to lower phase-locking at higher frequencies and periods of inhibition overlapping more with periods of excitation at higher frequencies. The corresponding best ITD, the FWHM, and the peak firing rate of the ITD tuning curves for the left MSO were quantified at 300 Hz and are shown in Figure 4.

The best ITD decreased linearly proportional to the myelin thickness of the SBC axon (Figures 4A,B). A positive best ITD value indicates that the left MSO fired more rapidly when the sound stimulus first arrived at the right ear. This result is in line with experimental observations and has been described by the opponent-channel coding model (Magezi and Krumbholz, 2010; Encke and Hemmert, 2018), where the ITD is encoded by contralateral MSO, for example, the right-leading ITD evokes the left MSO. In addition, as the myelin thickness increased (*d*_*SBC*_ > 0.35 μm), the best ITD value assumed values smaller than 0 ms. This shift caused the opponent-channel coding to fail and ITD responses as well as the coding scheme to reverse, predicting a sound location on the ipsilateral side of the brainstem. This result has not been experimentally reported. The SBC myelin thickness also altered the peak width (Figures 4D,E) and peak firing rate (Figures 4G,H) of the ITD tuning curve.

### Globular Bushy Cell Axon Myelin Thickness and Interaural Time Difference Tuning

An increase in myelination (*d*_*GBC*_) of the GBC axon along the contralateral inhibitory pathway (Figure 3E) showed significant changes to both the shapes and the scales of the ITD tuning curves with pure tone sound wave stimulation (Figures 3F–H). The best ITD value varied non-monotonically with GBC myelin thickness as shown in Figures 4A,C, where it first shifted toward the center (0 ITD), and later away from the center with a swing of more than 100 μs ITD (Figures 4A,C). The best ITD value reached its minimum when the GBC myelin thickness was around 0.25 μm and plateaued with a higher best ITD when the thickness was thicker than 0.3 μm. The peak width decreased when the GBC myelin thickness was increased, and subsequently became steady with thicker GBC myelination (Figures 4D,F). Note that a wider FWHM indicates broader tuned ITDs for the MSO, and a narrower FWHM suggests the tuning curve has a higher sensitivity and precision. Therefore, the results from this simulation indicate that GBC axons with relatively thicker myelination may yield more precise ITDs. Finally, the peak firing rate dropped substantially when the GBC myelin thickness increased from 0.2 to 0.3 μm, resulting in roughly stabilized peak firing rates (Figures 4G,I).

### Both Spherical Bushy Cell and Globular Bushy Cell Myelination Influence Interaural Time Difference Tuning

The interaction between myelin thickness of two contralateral inputs (one excitatory and one inhibitory) toward shaping ITD are shown in Figure 4. The increase of the SBC myelin thickness (*d*_*SBC*_) had a stronger effect on the best ITD, where the best ITD was increasing with thinner SBC myelin thickness (Figures 4A,B). Similarly, GBC myelin thickness (*d*_*GBC*_) also shifted the best ITD within a 100 μs range, especially for *d*_*GBC*_ from 0.2 to 0.3 μm (Figures 4A,C). The FWHM and peak firing rate were affected by both SBC and GBC myelin thickness. Thinner SBC myelin and thicker GBC myelin tended to produce narrower FWHM (Figures 4D–F) and lower peak firing rates (Figures 4G–I).

### Myelination Affecting Interaural Time Difference Encoding Accuracy

**FIGURE 5:**
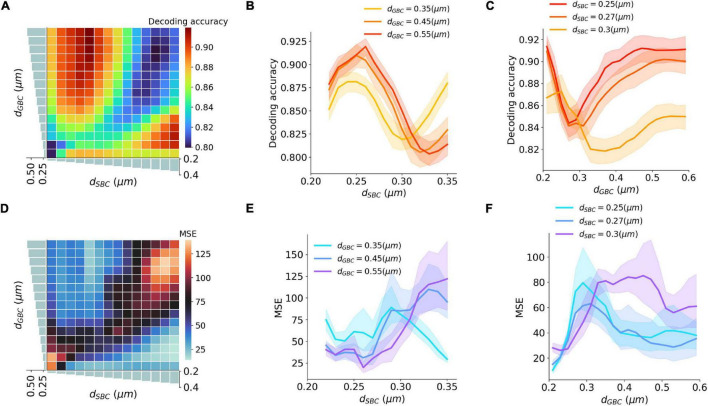
Interaction between SBC and GBC axon myelination on ITD decoding. **(A–C)** Decoding accuracy under different SBC and GBC myelin thicknesses. **(D–F)** Mean squared error (MSE) under different SBC and GBC myelin thicknesses. Error bands represent standard errors.

An ITD encoding analysis was conducted using population responses of MSO neurons to repeated pure tone stimuli with different ITDs. The encoding accuracy can be interpreted as the amount of ITD information extracted by the MSO (Figures 5A–C). The results indicate that the encoding accuracy reached its optimum when either SBC myelin was much thicker than GBC myelin, or when GBC myelin became much thicker than SBC myelin. The ability to accurately encode ITDs can also be represented as the mean squared error (MSE) between true ITDs and predicted ITDs (Figures 5D–F). Similar to the conclusion on ITD decoding accuracy, the contrast between SBC and GBC myelin thickness could result in smaller MSEs.

### Myelin Thickness and the Precision of Interaural Time Difference Decoding

**FIGURE 6:**
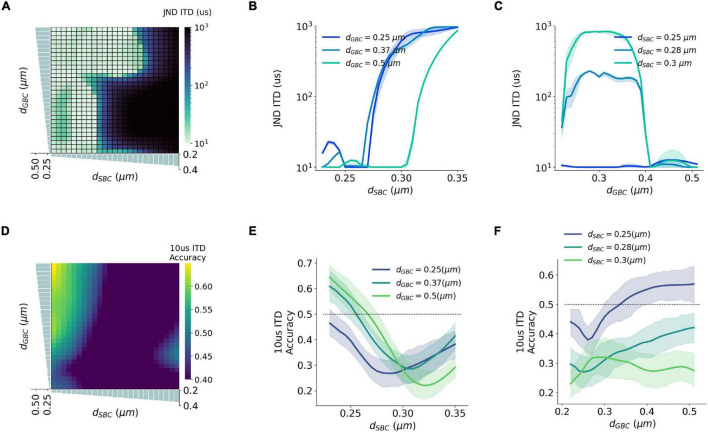
Interaction between SBC and GBC axon myelination on ITD sensitivity. **(A–C)** Just noticeable difference (JND) of ITD for different SBC and GBC myelin thicknesses. **(D–F)** Decoding accuracy of 10-us ITD for different SBC and GBC myelin thicknesses. Error bands represent standard errors.

Natural sound clips presented with small ITDs were utilized to stimulate the circuit and calculate the precision of ITD decoding. The just noticeable difference (JND) of ITD was calculated by comparing MSO responses to symmetrical ITDs, and the calculated JND can be regarded as the sensitivity to ITD stimuli. The best sensitivity was obtained when SBC myelin was thinner than 0.27 μm, or GBC myelin was thicker than 0.38 μm and at the same time SBC myelin was thinner than 0.3 μm (Figures 6A–C). Apart from this, the sensitivity became far worse when the best ITD approached zero. The worst sensitivity was obtained when the best ITD was negative. This result can be explained since the opponent-channel coding scheme was used in the JND calculation together with a one-tailed test. Besides calculating JND, the decoding accuracy in the range between 10 and –10 μs ITDs was computed to quantify the precision of the circuit to capture very small ITDs. The most precise accuracy was also acquired when the GBC myelin was much thicker than the SBC myelin (Figures 6D–F).

### The Effects of Contralateral Inhibition on Interaural Time Difference Decoding

**FIGURE 7:**
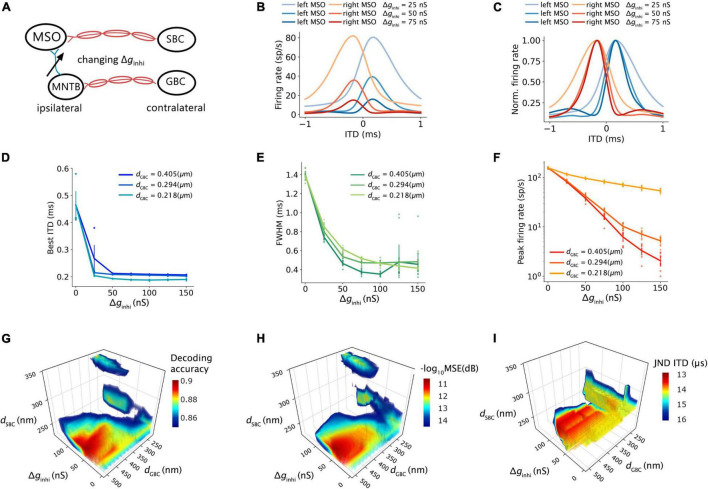
Effects of contralateral inhibition on ITD tuning and decoding. **(A)** Sketch describing tuned parameter. **(B,C)** ITD tuning curve **(B)** and normalized ITD tuning curve **(C)** as a function of different contralateral inhibitory synaptic strength. **(D)** Best ITD vs. contralateral inhibitory synaptic strength with an SBC myelin thickness of 0.248 um. **(E)** Full width at half maximum (FWHM) vs. contralateral inhibitory synaptic strength with the SBC myelin thickness of 0.248 um. **(F)** Peak firing rate vs. contralateral inhibitory synaptic strength with the SBC myelin thickness of 0.248 um. **(G–I)** Decoding accuracy **(G)**, mean square error in decibel **(H)**, and just noticeable difference of ITD **(I)** change with contralateral inhibitory synaptic strength and contralateral myelin thickness; values above 75% quantile were colored.

The SNN circuit was simulated with different synaptic strengths of the contralateral inhibitory inputs to estimate the effects of the contralateral inhibition on the ITD computation (Figure 7A). The shape of the ITD tuning curve changed as the inhibitory synaptic conductance (Δ*g*_*inhi*_) from MNTB to MSO increased (Figures 7B,C). The best ITD shifted closer to zero-ITD when the contralateral inhibition increased and reached a plateau with a Δ*g*_*inhi*_ above 25∼50 nS (Figure 7D). Both peak width and peak firing rate dropped with an increasing Δ*g*_*inhi*_ (Figures 7E,F). Combining the results with those obtained from varying inhibition strength and myelin thickness, the maximum decoding accuracy, the minimum MSE and the minimum JND could be obtained when the SNN circuit has an optimal Δ*g*_*inhi*_ of about 50 nS and the GBC myelin was 0.2 μm thicker than the SBC myelin (Figures 7G–I).

## Discussion and Conclusion

### Myelination, Conduction Velocity, and Spike Timing

In this study, the mammalian MSO circuit was modeled and tested with varying axon myelination properties to determine which role myelination plays in conduction velocity and transmission delay of action potentials. Physiological parameters included in our model were obtained from a number of published studies from Mongolian gerbils (Meriones unguiculatus), such that the resulting model should be a close representation of the gerbil.

We found that changes in axon myelin thickness affected the average propagation delays of action potentials between nuclei. A thicker myelin layer on GBC axons resulted in faster action potential propagation for the contralateral inhibition, and at the same time, a thinner myelin layer on the SBC axons resulted in longer delays for the contralateral excitation to MSO.

The model simulation demonstrated that the myelination of the contralateral input axons not only shifted the best ITDs but also influenced the peak firing rate and peak width of the ITD tuning curves, as shown in Figures 3, 4. This change in the shape of the ITD tuning curve was induced by the relative input timing of the excitation and inhibition, which controls the duration of the net post-synaptic potential and the time window of the binaural coincidence detection. For instance, when the time window became shorter, MSO neurons elicited fewer spikes during the sound stimulation period within a narrower ITD range, subsequently, its ITD tuning curve was altered toward a lower peak firing rate and a shorter peak width.

Our decoding results (Figures 5, 6) suggest that a larger GBC myelin layer combined with a smaller SBC myelin layer produced an optimal decoding accuracy and resulted in optimal ITD precision. In this scenario, action potentials associated with contralateral inhibition propagated faster than those associated with the excitation. This phenomenon had also been reported in previous experimental studies (Brand et al., 2002; Roberts et al., 2013). Specifically, it was postulated that MSO receives contralateral inhibition earlier than contralateral excitation, despite an additional synapse in the inhibitory pathway and a longer distance to cover. These findings may indicate that this tuning of myelination of axons on the contralateral pathways could be the consequence of structural adaptation of the sound localization pathway toward more accurate ITD detection.

It has been shown that myelination is fully established in the gerbils auditory brainstem significantly after hearing onset (Seidl and Rubel, 2016; Sinclair et al., 2017). Moreover, myelination patterns differ between axons responding to low vs. high frequency sound (Ford et al., 2015) and are altered when the animal’s sound experience is experimentally altered (Sinclair et al., 2017), suggesting that sound activity is involved in the establishment, and perhaps the maintenance of myelination. On the other hand, alterations in myelination as they occur in some conditions such as Fragile X (Lucas et al., 2021) or multiple sclerosis (Levine et al., 1993) or in animals with myelination deficits (Kim E. K. et al., 2013; Kim J. H. et al., 2013) result in temporally less well-timed activity in the sound localization pathway and/or impaired sound localization abilities, highlighting the functional consequences of these alterations. Taken together, these findings highlight the need for precisely controlled myelination patterns and suggest a possible mechanism to exert this control. The results of the present study are consistent with this body of work, highlighting how the computation of sound location in MSO changes with different myelination patterns.

### Negative Best Interaural Time Difference

The optimal decoding accuracy and small MSE can mathematically be also achieved with a completely opposite myelination pattern consisting of thicker SBC myelin and thinner GBC myelin (Figure 5). Under this opposite scheme, the peak firing rate became much higher, and the best ITDs were negatively shifted away from the zero-ITD (Figure 4). From a purely mathematical perspective, the higher peak firing rate achieved in this scheme increases the signal dynamic range, thus improving the signal-to-noise ratio for the encoded ITDs. The sign of the ITD can be thought of being irrelevant for the total amount of information, since negative or the positive best ITDs encode the same ITD value. However, from an experimental standpoint, such a non-opponent-channel coding scheme has not been observed.

### Possible Roles of Inhibition in Medial Superior Olive

Several competing models have been suggested for the role of inhibition in the MSO in ITD tuning (Brand et al., 2002; Pecka et al., 2008; Couchman et al., 2010; Grothe et al., 2010; Roberts et al., 2013; van der Heijden et al., 2013; Myoga et al., 2014; Franken et al., 2015). Some studies support the hypothesis that this inhibition modulates the peak timing of the excitation and tunes the coincidence detection. Notably, the pharmacological blockage of inhibition shifts the best ITD toward the zero-ITD (Brand et al., 2002; Pecka et al., 2008). Moreover, conductance clamp recordings (Myoga et al., 2014) demonstrated that precisely timed inhibition could tune the best ITD by modulating the net excitatory post-synaptic potential (EPSP), and the leading contralateral inhibition biased the coincidence detection timing about 50∼150 μs. The experimental result is comparable to our computational results (Figures 4A,C) in which the increased GBC myelin thickness modestly shifted the best ITD by only 100 μs but resulted in much improved sensitivity and decoding accuracy.

On the other hand, other studies have challenged this inhibition-tuning model. Although well-timed leading contralateral inhibition was observed in an *in vitro* study (Roberts et al., 2013), the ITD function and EPSP did not significantly differ with an inhibitory conductance of a 300-μs leading contralateral inhibition. In addition, although shift of best ITD toward zero was shown for pharmacological blockage, Roberts et al. (2013) described the role of inhibition as transient and less significant over time, inferring that the removal of inhibition should not systemically shift the best ITD (Franken et al., 2015). In these studies, the occurrence of inhibition decreased overall firing rates across ITDs and narrowed the ITD functions without shifting the best ITD. This trend is consistent with our results to some degree (Figure 7), as the best ITD was not shifted with varying inhibitory synaptic conductances unless the inhibitory conductance was lower than 50 nS (Figure 7D). The increased inhibition also reduced the peak firing rate (Figure 7E) and peak width (Figure 7F) in a way similar to the increased myelin thickness and conduction velocity on the contralateral inhibitory pathway (Figures 3E–H). On the other hand, we note that the ITDs produced by varying myelination in the afferent excitatory pathway exceed the biologically relevant range of at least most mammalian species (Figures 2B,C), while the smaller range of ITDs produced by varying myelination in the inhibitory pathway matches that range closer. It is, therefore, possible that this study underestimates that role.

The relative timing of the binaural excitation was concluded to be the dominant factor for ITD tuning due to its apparent capability to regulate the best ITD compared to the inhibition (Roberts et al., 2013; van der Heijden et al., 2013; Seidl and Rubel, 2016). However, through the decoding analysis, the linkage between the best ITD and the estimated ITD sensitivity was unexpectedly shown to be more indirect but in a profound way in which the best ITD shifts could not simply be used to predict the precision of the ITD detection. Even though the presence of the leading contralateral inhibition reduced the peak firing rate and narrowed the peak width of the ITD tuning curves, the decoding results revealed that the timing and synaptic strength of the contralateral inhibition largely attributed to the pinpoint precision and sensitivity of the ITD computation (Figures 7G–I). Therefore, our findings imply that the complexity of ITD tuning depends on the temporal interaction between the excitation and inhibition.

### Limitations

Our computational model was designed to probe the influence of axon myelination. We simplified the model and omitted several possible mechanisms of ITD encoding. First, a low-threshold potassium current shown to interact with the synaptic inhibition in MSO and sharpen the temporal sensitivity of the binaural integration (Khurana et al., 2011; Roberts et al., 2013; Myoga et al., 2014) was omitted. Second, post-inhibitory facilitation that can raise the firing rate under certain conditions (Beiderbeck et al., 2018; Ma et al., 2021) and had been observed in the MSO of juvenile mice (Dodla et al., 2006) was also not considered. This phenomenon could possibly compensate for a decreased firing rate induced by the leading contralateral inhibition in MSO. Third, a basic leaky integrate-and-fire model was utilized to improve computational efficiency and avoid some detailed physiological parameters, which have not been well characterized in gerbils. Nonetheless, the current model could be modified with the inclusion of diverse physiological parameters (Remme et al., 2014) to simulate rich membrane dynamics and temporal responses. Furthermore, besides spike conduction latency, spike conduction jitter in the auditory brainstem could affect precise temporal integration in sound localization (Reed et al., 2002; Marsalek and Kofranek, 2005; Kim E. K. et al., 2013; Kim J. H. et al., 2013). In our model, the conduction jitter was simplified as a constant 0.05 ms standard deviation that did not vary with myelin thickness.

### Conclusion

By using an SNN model of the auditory brainstem, we found that axon myelination regulated ITD computation. The myelination of contralateral excitatory pathways shifted the best ITD. Moreover, the myelination and synaptic strength of contralateral inhibition influenced the peak firing rate and width of the ITD tuning curve, and subsequently modulated the ITD precision and sensitivity.

## Data Availability Statement

The raw data supporting the conclusions of this article will be made available by the authors, without undue reservation.

## Author Contributions

B-ZL designed the study, conducted the study, analyzed the data, and wrote the manuscript. SP and MV designed the study. AK and TL designed the study and wrote the manuscript. All authors contributed to the article and approved the submitted version.

## Conflict of Interest

The authors declare that the research was conducted in the absence of any commercial or financial relationships that could be construed as a potential conflict of interest.

## Publisher’s Note

All claims expressed in this article are solely those of the authors and do not necessarily represent those of their affiliated organizations, or those of the publisher, the editors and the reviewers. Any product that may be evaluated in this article, or claim that may be made by its manufacturer, is not guaranteed or endorsed by the publisher.

## Footnotes

1
www.xeno-canto.org

2
https://github.com/AttwellLab/MyelinatedAxonModel/

3
https://github.com/libenzheng/MedialSuperiorOliveModel
